# Human Enhancement, Bioethics, and Moral Diversity: An International Philosophical Endeavor

**DOI:** 10.1093/jmp/jhag015

**Published:** 2026-09-21

**Authors:** Mark J Cherry, Ruiping Fan

**Affiliations:** Department of Philosophy, St. Edward’s University, Austin, Texas, USA; Department of Public and International Affairs, City University of Hong Kong, Hong Kong SAR

**Keywords:** biotechnology, genetic enhancement, moral diversity, moral enhancement

## Abstract

Human enhancement raises a host of important conceptual and moral questions drawn from ethics and metaphysics, social/political theory, and philosophy of medicine. What does it mean to be human? Is it permissible fundamentally to alter our basic humanity? What would that mean? Critics argue, for example, that it is morally permissible to engage in medical therapy to address disease and disability, but not biotechnology to enhance human capacities. Other detractors raise concerns that genetic enhancement would dehumanize persons, involve morally impermissible research, or serve objectionable social/political goals, violating basic human rights or human dignity. Supporters, however, have been known to contend that enhancement may be morally required to level the playing field, given an unequal natural distribution of genetic advantages, physical talents, and cognitive abilities. Others seek to rewire human freedom, biotechnologically engineering “morally improved” human choices or, perhaps, implanting computer chips in the brain, to avoid criminal behavior or international catastrophe. On whose account of morality would enhancements be objectionable or permissible? More generally, which bioethical norms should guide public law on such matters? This number of the Journal of Medicine and Philosophy brings together an international group of scholars from Hong Kong, Macao, Mainland China, the United States, and Texas, to explore critically such concerns regarding forms of human biotechnology, reasons for and objections to pursing various forms of enhancement, and the centrality of such philosophical questions to bioethics and public policy. Together, these authors present a conceptual geography of the possible meanings, creative realities, and likely limits of human bioenhancement.

## I. INTRODUCTION

The telos driving biotechnological development is deeply attached to the alteration, control, and advancement of human physical and mental capacities. Indeed, biotechnology has long enhanced perceived human deficiencies: artificial lenses to improve eyesight; cochlear implants and other aids to assist with hearing impairment; specialized chairs and computers to support mobility and communication; surgical practices and pharmaceuticals to treat the infirmities of aging, injury, and disease. Medical technology is utilized to improve muscle growth, increase height, sharpen mental focus, and enhance physical beauty. Innovations in genetic engineering, information science, and nanotechnology, however, may have brought mankind to the cusp of a major shift in genetic enhancement: augmentation of human nature itself (see, e.g., Tafazoli et al., 2019; Haleem et al., 2023; Modern Science Team, 2024). Gene-splicing techniques, such as CRISPR-Cas9, have made it much easier to “edit” the human genome—potentially removing genetic diseases or genetically altering basic human characteristics (see, e.g., Meisel, 2021; Laurent et al., 2024).

Human biotechnological efforts are no longer simply aimed at addressing an underlying pathology, but seek to augment and alter basic human physical features, cognitive abilities, and behaviors. Proponents claim that such enhancements permit intimate human and machine pairings so that the brain may interact directly with computers, allowing persons to upgrade their cognitive abilities or moral behavior, perhaps becoming conscious machines, or permitting those with physical disabilities better use of their arms, legs, and other body parts. Other developments aim genetically to engineer human beings to live indefinitely; to incorporate advantageous animal DNA, such as gills and webbed hands or feet; to adapt human beings for life in what are currently alien or lethal environments; to enhance strength and endurance; or to make human beings more resistant to deadly diseases, such as HIV/AIDS (Normile, 2019).

Human enhancement, however, raises a host of important conceptual and moral questions drawn from ethics and metaphysics, social/political theory, and philosophy of medicine (Bishop, 2020; Eberl and Ajibola, 2025; Xu, 2025). What does it mean to be human? Is it permissible fundamentally to alter our basic humanity? What would that mean? Some argue, for example, that it is morally permissible to engage in medical therapy to address disease and disability, but not biotechnology to enhance human capacities. Where “bio-liberals” tend to treat technological and genetic enhancements with unguarded optimism, so-called “bio-conservatives” often raise objections in principle, or encourage caution, regarding enhancement practices and trends. Supporters have argued that enhancement may be morally required to level the playing field, given an unequal natural distribution of genetic advantages, physical talents, and cognitive abilities. Other advocates seek to rewire human freedom, biotechnologically engineering “morally improved” human choices, or, perhaps, implanting computer chips in the brain. Detractors raise concerns that genetic enhancement would dehumanize persons, involve morally impermissible research, or serve objectionable social/political goals, violating basic human rights or human dignity. If such concerns are true, which enhancements would have such effects and why? On whose account of morality would enhancements be objectionable or impermissible? More generally, which bioethical norms should guide public policy on such matters?

This number of the *Journal of Medicine and Philosophy* brings together an international group of scholars from Hong Kong, Macao, Mainland China, the United States and Texas, critically exploring various forms of human bioenhancement. Ana Iltis, Carmen Harrell, Haley Tyrell, and Amaya Williams (2026) begin the issue with a detailed scoping review conceptualizing eight foundational areas of moral enhancement, comparing definitions, types and targets of interventions, reasons for and objections to pursuing enhancement, arguments regarding when enhancement is forbidden, permissible, or obligatory, as well as its implications for transhumanism, personal virtue, and the moral life.^1^ With this foundation in place, the issue turns to an examination of cross-cultural bioethical concerns. Ruiping Fan (2026) considers foundational distinctions among moral cultures and the ways different regions of the world may seek to address human genome manipulation. Jianhui Li and Yaming Li (2026) draw out a distinctively Confucian account of human dignity to explore permissible versus impermissible forms of bio-enhancement, while Ellen Zhang (2026) utilizes Daoism to critique technological alteration of the fundamental parameters of human existence. The next brace of essays assesses the meaning and possibility of moral enhancement. Xu (2026) explores such questions through Mencius’s Confucian theory of human nature, central to much thinking in China and the East; Jennifer Lockhart (2026) draws on Aristotelian thought to critique the possibility of artificially enhancing human virtue. The final paper turns the reader’s attention to the centrality of these philosophical questions to bioethics and public policy, as Mark J. Cherry (2026) engages with competing explanatory models of neurobiological determinism and human freedom to consider the implications of bioenhancement for political liberty. Together, these authors present a conceptual geography of the possible meanings, creative realities, and likely limits of human bioenhancement.

## II. HUMAN ENHANCEMENT: CONCEPTS AND DISTINCTIONS

Ana Iltis, Carmen Harrell, Haley Tyrell and Amaya Williams conceptualize the results of their detailed scoping review of the literature on human moral enhancement. They document the wide variety of terms, concepts, and distinctions on which authors rely to defend, reject, or explore moral enhancement. Basic terminology often varies: some authors refer to “enhancement,” others to “bioenhancement.” Indeed, even distinguishing between “enhancement” and “therapy” presents real challenges. As they note:

A particularly thorny question has been whether there is a difference between treatment and enhancement and, if so, whether a clear line may be drawn between them as well as whether the difference has any significance. Some people invoke the distinction as a way to distinguish morally permissible and impermissible interventions. Others use it to differentiate among interventions that a health professional ought to provide and those that such a person may provide but is not obligated to provide or, some would say, ought not to provide (Juengst, 1997).

The distinction also might be used to identify services that ought to be covered by third-party healthcare payers and those that should not. (Iltis et al., 2026, 402)

Definitions are often imprecise: some authors consider the term “enhancement” to identify advancements that push individual capacities beyond a “normal” human baseline (e.g., significant increases to human intelligence). Others prefer person-relative baselines, where “enhancement” refers to ways an individual might increase his own native capacities and performance (e.g., pharmaceuticals designed to improve athletic performance, mental concentration, or empathetic behavior). Sometimes concepts of “moral enhancement” reference specific goals, such as increasing prosocial behavior; others merely seek ways to improve moral behavior, without specifying any specific account of the right, good, virtuous, or just. Iltis et al. argue that common shared definitions, consistency of terminology usage, much less agreed-upon moral frameworks or moral conclusions, appear illusive. Their results reveal a complex scientific, social/political, and bioethical literature that is far from any sort of consensus.

As Iltis and her co-authors argue, the term “enhancement” offers a broad conceptual umbrella covering numerous types of interventions (e.g., genetic, pharmacological, technological, invasive interventions, noninvasive interventions, biomedical, non-biomedical, and so forth); targets of interventions (e.g., augmenting motives; improving moral behavior or morally relevant personal capacities, finding ways to increase the moral status of specific agents); as well as of the reasons for or goals of enhancement (e.g., to save humanity from significant harm, address urgent social problems, or fulfill duties to ourselves). They conceptualize a wide variety of objections to enhancement (e.g., risks of harm or threats to human agency, personal identity or human flourishing); as well as assessment of those conditions under which moral enhancement might be forbidden, permissible, or obligatory (e.g., some argue that enhancement is usually permissible if voluntary, others urge that it may be compulsory to reduce the risk of criminal activity). As they argue, this literature is rich and complex, drawing on numerous areas of philosophical concern, including assumptions regarding the nature and content of morality, the role of human intuitions, motivations, character, and virtue, as well as concerns about human free will versus determinism. Carefully teasing out such assumptions is essential if we are properly to critique and explore claims regarding human enhancement.

## III. REGIONAL VERSUS GLOBAL CONCERNS

As Iltis et al. demonstrate, the international scientific community, bioethicists, and lawmakers have long grappled with policy guidelines for regulating human enhancement. The empirical reality of quite different moral cultures and regional bioethical concerns significantly increases the difficulty of this task (Engelhardt, 1996; Cherry, 2024; Liao, 2025). Yet, as Ruiping Fan notes, all too frequently, international statements simply repeat content-full Western moral perspectives, as if such ethical assertions will (or ought to) be universally accepted (2026). Such analyses ignore the rich ethical diversity and moral complexities of regional and cultural perspectives on bioethics, such as the significant influence of Confucianism in East Asia. Fan argues that bioethical approaches that aim to be universal in scope must also be starkly limited in moral content. Moral analysis seeking relevance across the significant diversity of human cultures, societies, and religions will be able to offer, at best, minimal guidance. Fan concludes that so-called universal bioethical standards ought to recognize at most very minimal notions of basic human rights, such as forbearance rights against bodily harm and deception. Local cultures and regions, however, ought to be recognized as having authority to develop their own distinct and content-full bioethics–what Fan terms a “regional bioethical framework” (Fan, 2026). Such moral pluralism is just part of the human condition.

Ellen Zhang, Jianhui Li and Yaming Li provide two examples of a more regional bioethical approach. Ellen Zhang, for example, argues that while Daoism has a reputation for naturalism, this does not imply that it prohibits all forms of genetic engineering or advanced biotechnology for human enhancement. As Zhang concludes, “Daoist practitioners believe that technology can provide the means to enhance the physical and mental capacities of the human body and extend life beyond its biological limitations” (2026, 459). Indeed, Daoism has a centuries-long history of supporting the search for prolonging life or even achieving immortality. Human biotechnological enhancement may play an important role in this quest in regions of the world where Daoism plays a significant role in bioethics.

Li and Li, in turn, argue that for Confucianism genetic enhancement is not necessarily morally problematic. For example, “the aspiration to produce healthier offspring and eliminate genetic diseases aligns with Confucian ideals of fostering family prosperity and using human wisdom to fulfill Heaven’s design for human flourishing” (2026, 470). Individuals should harness and utilize their own agency to shape the environment, and themselves, in ways that support proper forms of human flourishing. Li and Li employ a specifically Confucian account of human dignity in support of their arguments: while all persons *qua human person* possess equal moral worth and potential (what they term “universal dignity”), individuals transform such moral capacities into forms of virtue through choice, activity, and commitment (what they term “personal dignity”). Genetic and other forms of enhancement may usefully address innate deficiencies in intellectual or physical abilities, creating more favorable conditions for the cultivation of individual virtues and enhanced personal dignity. However, there are limits:

the Confucian understanding of dignity—as rooted in respecting the essential boundaries of human nature established by Heaven—provides crucial ethical guidance for these technologies. Since dignity comes from living authentically within our Heaven-given nature rather than transcending it, both the development and application of genetic technologies must preserve the natural essence of humanity and must not fundamentally transform it. (2026, 470)

While genetic engineering to create human beings with wings or fur would undermine what it means to be human, they argue that remediating the harms of disease or enhancing central human capacities, such as intellectual ability and emotional connectedness, would likely support forms of personal dignity. As they note: “genetic interventions should restore or enhance our capacity to achieve authentic human excellence, rather than alter what it fundamentally means to be human (2026, 470). It is the active and intentional transformation of one’s innate abilities into appropriate Confucian virtues that leads to personal dignity. In support of Fan’s analysis, such Daoist and Confucian concerns may not have significant universal appeal; however, they likely resonate within large regions in the East and culturally similar parts of the world. In each case, the authors draw out substantial regional, rather than necessarily universal, ethical content to guide their analyzes.

## IV. GENETIC ENHANCEMENT AND THE ACQUISITION OF VIRTUE

The next two articles grapple with enhancement for the purpose of reshaping human nature: genetically modifying human beings for the acquisition of virtue. Hanhui Xu approaches these questions through Mencius’ theory of human nature; Jennifer Lockhart draws on Aristotle’s account of the physiological preconditions for acquiring virtue. While each concludes that direct genetic enhancement of moral virtue is not possible, both recognize that genetically modifying human nature so that it is easier to habituate persons to embody virtuous characteristics and decision-making may be permissible. In each case, the authors recognize that competing accounts of human behavior, causal determinism, and human freedom frame how we understand moral choice or what it means to embody virtue.

Xu, for example, argues that genetic enhancement aimed at improving empathic concern so that individuals are more inclined to help others fits within Mencius’ Confucian account of heart-mind, pity, and compassion. Confucianism tends to support moral perfectionism, he argues, “an attitude toward human perfectibility–an attitude of ‘incessant conversion’ to one’s more perfect self” (2026, 479). We are to strive to reach ideal moral character and “noble personhood” (2026, 479), which requires commitment to personal growth, cultivation of virtue, expansion of knowledge, and refinement of character. Insofar as genetic enhancement would improve one’s feelings of empathy, making it more likely that one will become a virtuous person, and the world a more peaceful place—that is, helping persons orient toward moral perfectionism—it would be compatible with Confucianism. However, one must be cautious; it would be deeply problematic to modify human empathy in ways that would undermine central Confucian interests in filial piety or concern for one’s own well-being. Augmenting or altering human nature must fit within traditional Confucian concerns for the family and human flourishing.

In many ways, Lockhart’s exploration of Aristotelian virtue comes to similar conclusions. She argues, for example, that there are at least two central problems for attempting to enhance human virtue though biotechnology.

1. *Habituation:* Virtues of character are acquired through habituation, a process of ethical upbringing or formation that involves the repetition of certain actions accompanied by the appropriate feelings of pleasure and pain and reinforced by rational discussion of the meaning and appropriateness of these feelings and choices.

2. *Judgment:* Prudential wisdom is a necessary condition for virtue since virtues of character require the capacity to judge what is appropriate and to feel and to choose in light of the particularities of the situation. (2026, 487)

Aristotelian virtue requires active character formation and rational participation in prudential judgment, which is acquired through practice and habituation; it is not acquired by nature. So, attempting to encode virtue into the human genome simply misses the point. As Lockhart notes: “The expertly trained sentiment and judgement that are the markers of the virtuous person are acquired by being brought up in a particular way. By repeatedly performing certain sort[s] of actions and learning to understand them and to take pleasure in them, one becomes a good person” (2026, 487). Even if it were possible to genetically engineer human beings who act in accordance with virtue and the good, they would not be virtuous individuals or good persons. Indeed, “They would be quite defective people, unable to cope with language or culture” (2026, 487).

Human beings, born defenseless, without locomotion, with large brains, and reared for many years, make their way by being *able to be habituated*— they are able to be brought up into a particular conventional culture. This means that human excellence when in full flower will rely on more than proper physiology (that might be encoded in genes). It will be necessarily beholden to good upbringing. The plasticity necessary for so being brought up is itself constitutive of the kind of excellence that becomes embodied by human beings. (2026, 488)

Genetic engineering, or other forms of bioenhancement, to modify natural human physiology so that people are more easily habituated into virtue might fit within Aristotle’s account of virtue as a form of cultivated excellence. Human physiology cannot bring about virtue on its own; parents, educators, and lawmakers are essential for educating prudential judgment and inculcating virtuous habits into persons. Human striving will always be necessary.

## V. CONCLUDING THOUGHTS

Insofar as biotechnology makes it possible to enhance human abilities, conquer genetic frailties, engineer moral persons, or even create a human biology that is physiologically more malleable to being habituated to virtue—attempting to improve on human nature or alter human beings in line with specific cultural or social desires—such temptations would carry significant ambiguities and risks. In part, as Cherry (2026) argues, and the articles in this issue illustrate, moral diversity is deeply embedded in the human condition. There is no general agreement regarding the nature of the right, good, virtuous, or just. Instead, there are numerous competing accounts of the ethically reasonable, morally rational, or personally virtuous, each with its own set of normative intuitions and metaethical theory designed to secure some specific understanding of the moral project. The underlying values and classification schemes that frame ethical judgments change over time, varying among cultures, places, and points in history. What is judged virtuous and good, pro-social behavior, rather than inappropriately antisocial or unduly risk-affirming, generally depends on who is doing the judging and the choice of normative criteria. Not all persons are risk averse; moreover, it is not obvious when risk-affirming activity necessarily crosses the line into immoral behavior. As Cherry (2026) notes, the annual costs to society from socially sanctioned but potentially risky activities, such as sexual promiscuity, youth sports, traveling by automobile, and alcohol consumption, are significant.

In part also, as Cherry argues, technologically “repairing,” pharmaceutically altering, or genetically redesigning brain circuits to correct for perceived antisocial behavior may lead to more harm than benefit. As he notes, such “personal characteristics are frequently useful: consider the important roles of soldiers and high-rise construction or explosive demolition workers, as well as risky entrepreneurial pharmaceutical or industrial development” (Cherry, 2026, 504). Bioengineering human nature to be more easily habituated to virtue may just make it more straightforward for authoritarian political elements to take advantage of a weakened and more easily manipulated population. Those who call for such governmental interventions seem to assume that state actors will be fully justified in imposing the advocates’ own preferred moral point of view, even though the significant diversity of moral and political perspectives is easily documented (Cherry, 2024).^2^ Indeed, as Cherry concludes: “Those in positions of authority should be prevented from seeking to punish (or reprogram) political opponents or citizens who express different moral/political points of view” (2026, 504). Consequently, he calls for significant caution in such matters, since seeking to shift human behavior through medical interventions has all too often manifested as a convenient tool for political oppression.

Such concerns are not unique to human enhancement. Similar ethical, metaphysical, and social/political concerns arise in the context of biotechnological developments seeking to perform head/brain transplants,^3^ create artificial wombs,^4^ support forms of assisted dying and other interventions at the end of life,^5^ unite human beings with artificially intelligent computer interfaces, or engage in radical body transformations,^6^ for example. While bioethics as a field of inquiry often aspires to a universal account of morality to guide scientific development and public policy, adequate attention to the significant challenges that religious, cultural, and secular moral diversity presents to the ethical assessment of new and innovative forms of biotechnological development is essential. The contributors to this special thematic issue often do not agree regarding the nature and ground of bioethics, which forms of human excellence or moral virtue should be encouraged, much less when genetic or other forms of biotechnological enhancement would be forbidden, permissible, or obligatory. However, as each author makes clear, careful, conceptual, philosophical analysis is essential for exploring the moral, social/political, and metaphysical issues at stake. How different societies, global regions, cultures, or religions come to terms with such conceptual, moral, and social/political concerns will have a significant impact on the future of medicine and culture. It is our honor to present this ongoing discussion as part of the *Journal of Medicine and Philosophy.*

## Notes

1 For more insight into concerns of transhumanism and the post-human, see Chavez (2025), Parviz (2025), Eberl (2025), Hershenov (2025), Bishop (2025), and Debates (2025).

2 Here, one might consider the wide variety of religious perspective on such issues; see Pilkington (2025), Tarabrin and Tarabrin (2025), Hogan (2025), Tran and Crawford (2025), Sportiello (2025), Delaney (2025).

3 For detailed explorations of the possibility of head/brain transplants, see Cherry and Fan (2022), Cherry (2022), Eberl (2022), Bian and Fan (2022), Iltis (2022), and Parker (2022).

4 The development of successful artificial wombs will raise significant new questions for the abortion debate. See, for example, Gross (2024), Roger and Blackshaw (2024), Anderson (2024), and Colgrove (2024).

5 See, for example, Richards (2025), Tzinman (2025), Farrelly-Jackson (2024), and LiPuma and Demarco (2024).

6 See, for example, Moeller and Ponseti (2025) and Dierckxsens and Baron (2024).
